# Circumstantial evidence for an increase in the total number and activity of borrelia-infected *ixodes ricinus* in the Netherlands

**DOI:** 10.1186/1756-3305-5-294

**Published:** 2012-12-17

**Authors:** Hein Sprong, Agnetha Hofhuis, Fedor Gassner, Willem Takken, Frans Jacobs, Arnold JH van Vliet, Marijn van Ballegooijen, Joke van der Giessen, Katsuhisa Takumi

**Affiliations:** 1 Centre for Infectious Disease Control, National Institute for Public Health and the Environment (RIVM), Bilthoven, The Netherlands; 2 Laboratory of Entomology, Wageningen University, Wageningen, The Netherlands; 3 Environmental Systems Analyses Group, Wageningen University, Wageningen, The Netherlands

**Keywords:** *Borrelia burgdorferi* sensu lato, *Ixodes ricinus*, *Population dynamics*, Lyme disease; The Netherlands

## Abstract

**Background:**

Between 1994 and 2009, a threefold increase has been observed in consultations of general practitioners for tick bites and Lyme disease in The Netherlands. The objective of this study was to determine whether an increase in the number of questing ticks infected with *B. burgdorferi* sensu lato is a potential cause of the rise in Lyme disease incidence.

**Methods:**

Historic data on land usage, temperature and wildlife populations were collected and analyzed together with data from two longitudinal field studies on density of questing ticks. Effective population sizes of *Borrelia burgdorferi* s.l. were calculated.

**Results:**

Long-term trend analyses indicated that the length of the annual tick questing season increased as well as the surface area of tick-suitable habitats in The Netherlands. The overall abundances of feeding and reproductive hosts also increased. Mathematical analysis of the data from the field studies demonstrated an increase in mean densities/activities of questing ticks, particularly of larvae between 2006 and 2009. No increase in infection rate of ticks with *Borrelia burgdorferi* sensu lato was found. Population genetic analysis of the collected Borrelia species points to an increase in *B. afzelii* and *B. garinii* populations.

**Conclusions:**

Together, these findings indicate an increase in the total number of Borrelia-infected ticks, providing circumstantial evidence for an increase in the risk of acquiring a bite of a tick infected with *B. burgdorferi* s.l. Due to the high spatiotemporal variation of tick densities/activities, long-term longitudinal studies on population dynamics of *I. ricinus* are necessary to observe significant trends.

## Background

Lyme disease, or Lyme borreliosis, is the most prevalent tick-borne infection of humans. In the early stages, Lyme disease is clinically manifested by an erythema migrans, an expanding skin lesion occurring after several days or weeks at the site of the tick bite. Late and more serious Lyme disease is a multi-system disorder with skin, neurological, cardiac and musculoskeletal manifestations [1,2]. Over the last decade, the incidence of Lyme disease has increased in at least nine European countries [3]. In The Netherlands, a long-term retrospective study among all general practitioners (GP) has shown a strong and continuing increase in GP consultations for erythema migrans during the past fifteen years from 4 per 10,000 inhabitants in 1994 up to 13 per 10,000 inhabitants in 2009 [4,5]. A similar magnitude of increase was observed in the number of people that reported a tick bite: The incidence of GP consultations for tick bites was 19 per 10,000 inhabitants in 1994, and increased to 56 tick bites per 10,000 inhabitants in 2009 [4,5]. The most straightforward explanation for the increase in Lyme disease is therefore the increase in the exposure to infected ticks. Despite considerable efforts during the past decades in education of the public, aiming to reduce human exposure to ticks and promote timely removal of ticks from the human skin, the rise in the incidence of Lyme disease continued.

The risk of acquiring Lyme disease depends on many different biological, environmental, and societal factors [6-14]. In short, however, it depends on two main factors: the abundance of questing *Ixodes ricinus* ticks infected with the causal agent *B. burgdorferi* sensu lato (s.l.), and the level of human exposure to ticks. In this study, we assessed whether the number of questing ticks infected with *B. burgdorferi* s.l. in The Netherlands has increased during the past decades. Besides direct methods such as tick sampling from the field, we explore several indirect measures to investigate longitudinal trends in the number of questing Borrelia-infected nymphs.

The number of questing ticks in an area is determined by the tick density and their level of activity, which, in turn, is determined by a complex interplay between vegetation, climatic conditions and the presence of blood hosts. For example, vegetation provides questing sites for ticks, but it also affects micro-climatic conditions, such as humidity and temperature, which determines tick survival and activity [15-18]. Vegetation also influences utilization by host animals and affects questing times by providing different degrees of shelter for ticks. An increase in tick bites may be due to an increase in tick density or activity but could also be due to an increase of tick suitable areas or exposure.

Temporal weather and climate conditions appear to be predictors for the tick activity, and to a lesser extent tick density [19-22]. Nymphal and adult ticks tend to quest for a blood meal once the weekly mean daily maximum temperature exceeds 7°C [23-25]. The development of ticks depends upon the consumption of vertebrate blood. Therefore, the abundance of feeding hosts can affect the abundance of ticks. All mobile life stages of *Ixodes ricinus* can feed on a broad range of warm- and cold-blooded vertebrate hosts [12,21,26,27]. *Ixodes ricinus* larvae infest small mammals, but also feed on larger animals such as roe deer. Nymphs and adults usually feed on medium-sized and large mammals [28]. These differences are probably due to the differential vertical distribution of instars [29]. To investigate trends in the relative abundance of blood hosts during the past decades, we analyzed readily available data on the abundance of roe deer and fallow deer, birds, rodents, and proxies for the abundance of rodents such as birds of prey [30,31].

The best available estimate for the tick density is the activity of questing ticks measured by standard blanket dragging, despite the limitation that moulting, resting and feeding ticks are not caught [32,33]. Furthermore, this technique is not equally efficient in different vegetation types, and therefore will not provide absolute numbers of questing ticks from a given area. Measuring the number of ticks searching for a blood meal, and testing these ticks for infection with *B. burgdorferi* s.l. provides an estimate of local and temporal variation in public health risk. To search for longitudinal trends in the number of questing ticks and tick infection with *B. burgdorferi* s.l., tick sampling data from two field studies was analyzed. Our analyses involved a long-term field study at a single location [18,34,35], and an ongoing longitudinal field study at thirteen locations geographically spread throughout The Netherlands [18].

Finally, we applied a population genetic approach as a complementary indirect exploration for changes in the abundance of *B. burgdorferi* s.l. infected ticks that were collected in these field studies. For this purpose, we used genetic information of Borrelia-DNA found in ticks to construct phylogenetic trees, mapping the genetic divergence within and between *B. burgdorferi* genospecies. Using this reconstruction of the recent demographic history of *B. burgdorferi*, we looked for indications of changes in the genetic diversity over time, which may suggest changes in the sylvatic transmission of *B. burgdorferi* s.l. in The Netherlands.

## Methods

### Surface of tick suitable areas

A database of land usage in The Netherlands (LGN) was used as a primary data source (Alterra, Wageningen). The total area of each land type during the periods 1999–2000, 2003 – 2004 and 2007–2008 was extracted from LGN using ArcGIS 9.3 (ESRI: Redlands CA). Resolution of the map was 25m by 25m. The country was partitioned into 42 land types in LGN. To each of the 42 land types, we assigned one of three new labels indicating habitats with probabilities for a high, low, or zero-tick density (see Additional file 1: Table S1). These values are partially based on several (ongoing) nationwide surveys [18,34,36]. Forests, dunes, and natural grasslands were assigned the category ‘high tick density area’. Pastures, orchards, tree(s) and grass patches *within* (sub) urban areas, bog and peat area, and heather were assigned the categories ‘low tick density area’. Agricultural area, (sub) urban areas, motor and railways were assigned the category ‘zero tick density area’. The total area for each of the three habitats was calculated by adding the areas of corresponding land types.

### Length of tick questing activity season

Mean daily temperatures at the weather station in De Bilt during 1985–2010 were extracted from the online database (Wolfram Research, Inc., Champaign, IL). The number of days above 7°C was counted annually and a linear trend was assessed using a generalized linear model for Poisson counts. Data points are shown in Additional file 2: Figure S1. The analysis was repeated using only the days above 7°C of the first half year (January until June), to focus more on the onset of the annual tick questing activity and to avoid the inhibitory effects of the shortening day length in the autumn on the questing activity of ticks [24].

### Abundance of vertebrate hosts: birds and small rodents

We analyzed a database containing a total of 215 distinct species of birds that were monitored across the country between 1990 and 2008 (Netwerk Ecologische Monitoring, SOVON&CBS, http://www.sovon.nl). Sixty-five species of winter birds and twenty species of water birds were excluded from the analysis because they were deemed less relevant for the sylvatic transmission of *Borrelia*[37]. Population index values for the remaining group of 130 bird species were described by the negative binomial distribution, where the annual mean index value is an exponential function in time with the rate r. The presence (r > 0) or the absence (r = 0) of trends in the population index values in time was assessed by the likelihood ratio test. Lacking direct measurements of rodent populations, we selected bird of prey species as indicator species for a trend in the rodent population densities on a nationwide scale [30,31]. Barn owl (*Tyto alba*) and common buzzard (*Buteo buteo*) are birds of prey that specialize on mice and other small rodent species, and they are distributed widely in The Netherlands. A trend in these bird population indexes was assessed as described for other bird species.

### Abundance of vertebrate hosts: roe deer and fallow deer

Roe deer (*Capreolus capreolus*) are especially important for maintaining (and determining the level of) tick populations because they feed reproductive females [22]. After hatching and diapause, the larvae become active the following spring [38]. In Western Europe, roe deer is one of the most abundant propagation hosts of *I. ricinus*[39,40]. To detect a possible association between roe deer population density and tick abundance in the following year, the trend in national population density of roe deer in 1980, 2003, 2004, and 2008 (Dutch Royal Hunting Society, KNJV) was analyzed using a generalized linear model. For the spatial correlation analysis, location-specific roe deer population density estimates (in 2008) at the tick-sampling sites were extracted from the roe deer database and paired with the estimated numbers of questing ticks of all stages in 2009. Data on roe deer and fallow deer (*Dama dama*) abundance from Duin and Kruidberg, a coastal dune area, were available from the wildlife management unit in Noord Holland [41]. Correlation between roe deer and questing ticks was assessed using generalized linear models with a gamma distribution and the negative binomial link.

### Data on tick densities/activities and infection rate

Data on tick densities/activities were derived from two large field studies conducted in The Netherlands [18,34,35]. In one study, ticks were collected by standard blanket dragging at thirteen locations geographically spread throughout the country from 2006 to 2009 at monthly intervals [18]. Criteria for selection of these study areas were based on the likelihood of finding any *I. ricinus* ticks, and the availability of a team of trained volunteers to make monthly blanket drag collections [18]. In a separate longitudinal study, a single area in the coastal dunes, Duin and Kruidberg, between 2000 and 2009 was selected at that time because of its unusually high tick density/activity. This area is rich in vegetation and open to the public for recreation [18,34,35]. Monthly tick collections were performed from April to October. For the distribution of the study areas in The Netherlands, see Additional file 3: Figure S2.

Seven genospecies of *Borrelia burgdorferi* s.l. known to circulate in Europe have previously been identified in The Netherlands by reverse line blot analysis [34]. The prevalence of *Borrelia* genospecies was determined by PCR followed by reverse line blotting. DNA sequences of the variable 5S-23S intergenic spacer region were determined from 211 randomly selected *B. burgdorferi* sensu lato isolates from infected nymphs (98%) and adults (2%), as described by Wielinga *et al.*[34]. These genospecies were categorized by previously defined reference sequences [42]. *Borrelia bavariensis*, a recently defined genospecies, was also found by sequencing some *B. garinii* isolates. As our Reverse Line Blot cannot distinguish between *B. garinii* and *B. bavariensis*, little can be inferred for the prevalence of this newly named genospecies in The Netherlands.

### A model for the numbers of questing ticks

Numbers of questing ticks are highly variable measurements. Hence, a model describing a tick sampling process is necessary to make inference about a multi-year trend in tick densities. The variation in questing tick densities was greater than Poisson. Thus, the probability that *i*-ticks are collected at a particular study site on a particular sampling time was empirically shown by the negative binomial distribution [43,44].

$$f\left(i\right)=\frac{\left(k+i-1\right)}{i!\left(k-1\right)!}{\left(1+\frac{m}{k}\right)}^{-k-i}{\left(\frac{m}{k}\right)}^{i}$$

The parameter *m* is the mean number of questing ticks per study site. The parameter *k* is a measure of aggregation where a highly aggregated number of questing ticks is indicated by a small value of *k*. Seasonality and other location-specific conditions are likely sources of variations in the numbers of questing ticks. Thus, we wrote the mean (*m*) as a product of the seasonal variation (*s*) and a multi-year trend (*u*)

$$m\left(t\right)=u\left(t\right)s\left(t\right)$$

The time (*t*) is expressed as a fraction of the sampling period where one year was scaled to a period of 2π. Seasonality is a trigonometric function

$$s\left(t\right)=1-\mathrm{Cos}\left(t-\tau \right)$$

The mean number of questing ticks is therefore periodic and it reaches the maximum questing activity when *t* = *π* + *τ*. The multi-year trend is the linear function

$$u\left(t\right)=a+\mathrm{bt}$$

The parameter *a*, is the mean tick density/activity in the baseline year per drag area. The parameter *b* is the change in tick density/activity per time unit.

#### Parameter estimates

Numbers of ticks captured at different times at the study sites were used to estimate the parameters of the model; annual mean number of questing ticks (*a*), multi-year trend (*b*), the moment of peak questing activity (τ), and the parameter *k*. The set of parameters was estimated specifically for each study site by maximizing the log-likelihood of the questing tick counts. Probability (*P*-values) that the null hypothesis of a constant tick density/activity is the correct model (i.e. whether *b* = 0) was calculated for each study site by likelihood ratio. *P*-values less than or equal to 0.05 were considered to be a significant support for the model describing the multi-year trend.

#### The annual total estimated abundance of questing ticks

The total number of questing ticks in one year was calculated by integrating the estimated tick activity function over one sampling year for each site, and subsequently averaging the resulting total numbers over the study sites. The overall change during the sampling period was calculated by dividing the total annual tick activity for 2009 by the total annual tick activity for 2006.

### Trends in the prevalence of ticks with *B. burgdorferi* sensu lato

Nymphal and adult ticks, collected from the field studies, were tested for the presence of *B. burgdorferi* by PCR followed by reverse line blotting. Numbers of tested ticks were highly variable. To remove effects of variable sampling sizes on the estimate for the prevalence, the numbers of questing infected ticks were described by the binomial distribution. The probability that a tick is positive for *B. burgdorferi* was modeled by the logit function.

$$p=\frac{e^{z}}{1+e^{z}}$$

To test seasonality, we set *z* = *x* + *yCos*(*t* − *θ*) where time *t* is expressed as a fraction of the sampling period where one year was scaled to 2π. To test for site-specific prevalence, we determined two clusters of the study sites using the squared Euclidean distance of the raw prevalence values. The parameters *x*, *y* and θ were estimated by maximizing the likelihood of observed numbers of ticks with *B. burgdorferi*. Using the likelihood ratio we assessed whether all study sites are characterized by the same seasonal oscillation or one cluster of study sites was characterized by higher prevalence values than the other cluster.

### Population dynamics of *Borrelia burgdorferi* s.l

The DNA sequences of the intergenic 5s-23S spacer region from *Borrelia* isolates derived from questing ticks were used to estimate changes in the effective population size of infected ticks. This analysis was limited to the two main Borrelia genospecies for which a sufficient number of samples were available: *B. afzelii* (n=86) and *B. garinii* (n=60). Note that in these two cases the number of samples is relatively small for coalescent analyses [45]. We excluded the so-called *B. ruskii* subclade from the *B. afzelii* samples, because of the unusual sequence divergence between the -ruskii and the other -afzelii samples (see Additional file 4: Figure S4).

We tested changes of genetic diversity of Borrelia over time by calculating Fu’s Fs statistics using Arlequin v.3.5 [46]. A large negative value of Fs indicates an excess of rare alleles, as would be expected from a recent population expansion or from genetic hitchhiking [47]. We also used the software Beast for Bayesian analysis of molecular sequences [48] to estimate coalescent effective population size with skyline plots [45]. Skyline plots represent a nonparametric flexible method to estimate changes in the coalescent effective population size, and here it is used as a measure for changes in genetic diversity, which is complementary to Fu’s Fs. We used the general time reversible model of DNA evolution with site specific mutation rates (gamma distribution) and default priors. Simulations were run for 30 million updates after discarding burn-in.

## Results

### Increase in surface of tick suitable areas

Between 2000 and 2008, habitats suitable for high-tick densities increased in surface area by 19% (+847 km^2^, Table 1), predominantly by expansion of forest areas. Low-tick density habitats increased only marginally in area (Table 1). Zero-tick density habitats decreased by 8% (−1192 km^2^, Table 1).

**Table 1:** Areas in The Netherlands in 2000 – 2008 categorized according to three levels of probable tick densities

**Density**	**2000**	**2004**	**2008**	**Change 2000-2008**
High	4399 km^2^	4483 km^2^	5246 km^2^	19%
Low	15608 km^2^	15517 km^2^	15645 km^2^	1%
Zero	13744 km^2^	13755 km^2^	12652 km^2^	−8%

### Length of tick questing activity

The mean number of days above 7°C (the temperature above which nymphal and adult ticks become active) has increased linearly with the year since 1985 from 235 to 262 days in 2009 (P-value = 0.01, see also Additional file 2: Figure S1A). To exclude the adverse effects of the shortening day length in the autumn on the questing activity of ticks [24], the analysis was repeated using only the days above 7°C of the first half year (January until June). Between the years 1985 and 2009, the number of days in the first half of the year when the average daily temperature exceeded 7 degrees Celsius increased significantly (p = 0.002) from 96 days in 1985 to 118 days in 2009 (see also Additional file 2: Figure S1B). Changing the threshold temperature to 10°C (the temperature above which larval ticks also become active), the mean number of days above 10°C has increased linearly with the year since 1985 from 177 to 204 days in 2009 (P-value = 0.003). This proxy for the tick questing season length has been increasing since 1985. Trends in reported tick bites and erythema migrans have only been available since 1994. In the shorter time window of 1994 – 2009, only the mean number of days above 10°C significantly increased.

### Abundance of birds and birds of prey as proxy for density of small rodents

**Figure 1:**
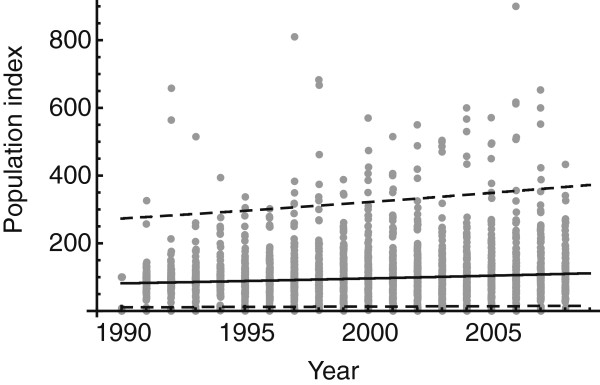
**Bird population densities 1980–2008.** Dots represent population index values of each bird species per year. The bird population index was calculated by dividing the number of a bird species each year by the number of that bird species in the baseline year in 1990 and multiplying the result by 100. Lines represent the best-fit negative binomial distribution with the mean (solid) and the 95% confidence interval (dashed).

The analysis of the longitudinal bird records revealed that the pooled populations of breeding birds increased significantly during the period 1990 – 2008 (P-value < 0.001, Figure 1). In 2008, there were 1.4 birds for each breeding bird in the baseline year, 1990, i.e. a 40% increase (Figure 1). This could be an indication that the tick host availability, and therefore feeding capacity, has increased during the past twenty years.

**Figure 2:**
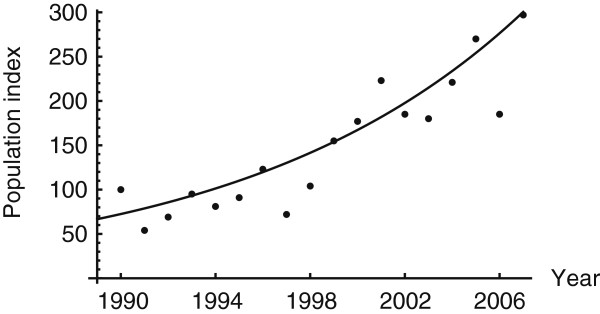
**Change in the number of Barn owl (*Tyto alba*) 1990 – 2006.** The change in the number of Barn owl was calculated by dividing the number of barn owls each year by the number in the baseline year 1990 and multiplying the result by 100. The solid line is the best fit exponential function.

Since 1990, the barn owl population index in the country has increased at the rate of 0.084 birds per year (*P*-Value < 0.0001) (Figure 2). The common buzzard population in the country has also increased during 1990–2007 at the rate of 0.033 birds per year (P-value < 0.001). As populations of birds of prey are assumed to be correlated with rodent abundance [30,31], these data suggest that the abundance of rodents increased between 1990 and 2007.

### Abundance of roe deer

**Figure 3:**
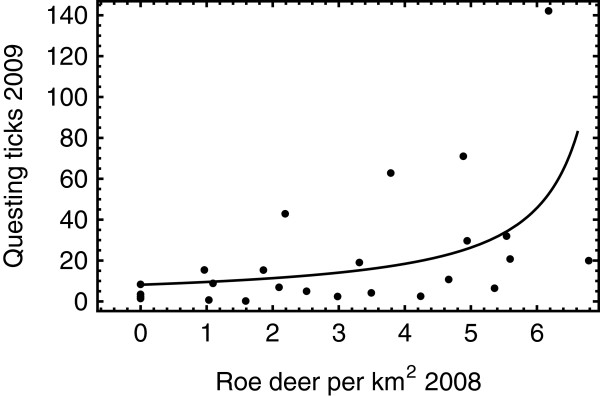
**Roe deer population densities and questing tick abundance.** Horizontal axis is the local population density of Roe deer at all fourteen study sites in 2008. Vertical axis is the peak number of questing ticks at the corresponding study sites in 2009. The solid line is the best generalized-linear-model fit.

The estimated nationwide population size of roe deer in The Netherlands has increased linearly between 1980 and 2008 (*P*-value = 0. 0009), growing each year on average by 1820 animals to ca. 70,000 animals in 2008. Roe deer population densities at the fourteen tick-sampling sites in 2008 were associated significantly (P-value = 0.005) with the numbers of questing ticks of all stages at the corresponding sites in the following year (Figure 3). When we tested the same association in the 2-year period between 2004 and 2006 at the fourteen sites, the association was significant as well (P-value = 0.008). An increase over low deer densities (0–3) has rather little effect on tick abundance until the upper end of (>5) the range of deer density (Figure 3). Thus, the increasing availability of blood meal sources is positively associated with tick reproduction (natality), host finding or tick survival.

### Tick density/activity in the 13 study areas

All ticks that were collected at the thirteen study sites (Additional file 3: Figure S2) in monthly intervals from 2006 to 2009, were identified to be *I. ricinus*[18]. By applying an empirical model-based statistical analysis to each study site, we detected significant three-year trends in the density/activity of questing ticks at six study sites (Table 2). The cumulative number of questing larvae, nymphs, and adult ticks increased in density/activity at five of these study sites and decreased at one other site. A three-year trend was absent at the remaining seven sites (Table 2). The estimated overall density/activity of questing ticks increased by 21% during the three-year period (2006 – 2009). The number of nymphs and adult ticks increased significantly only at two study sites and decreased at one site (Table 3). At the remaining ten study sites, significant trends in tick density/activity were not observed. The estimated overall density/activity of questing nymphs and adults increased by 4% during the same period. Hence, the increase in the total tick density/activity was mainly due to questing larval ticks.

**Table 2:** **Estimates for the parameters in the model describing the densities of questing larval, nymphal and adult *Ixodes ricinus *2006 – 2009**

**Sampling locations**	**Baseline tick density/activity**	**Population trend**	**Month of peak tick density/activity**	**Tick aggregation**	**P-value**
**Appelscha**	**1.5**	**1.5**	**6.1**	**2.7**	**<0.01**
Bilthoven	3.4	0.0	6.7	1.4	0.07
**Ede**	**45.0**	**11.9**	**6.4**	**1.1**	**0.05**
Eijsden	10.7	0.0	6.1	1.1	0.08
**Gieten**	**5.5**	**18.4**	**6.2**	**0.5**	**<0.01**
**Hoog Baarlo**	**67.4**	**−11.4**	**6.6**	**1.1**	**<0.01**
Kwade hoek	3.5	0.0	6.4	0.4	1.00
Montferland	25.2	0.0	6.7	0.4	0.09
**Schiermonnikoog**	**1.5**	**3.4**	**6.8**	**0.5**	**0.02**
Twiske	22.0	0.0	6.0	0.7	0.30
Vaals	10.0	0.0	6.5	0.2	0.64
Veldhoven	32.8	0.0	6.7	0.7	0.11
**Wassenaar**	**0.0**	**6.2**	**5.8**	**0.4**	**<0.01**

Increasing trends are indicated by positive estimates for the coefficient *b* in the linear function (Experimental procedures), decreasing trends by negative estimates. Baseline is equal to the intercept *a* in the linear function describing the tick density/activity per drag area. Population trend is the change in tick density/activity per year; it is equal to the coefficient *b* in the linear function multiplied by 2π. Peak month is equal to the parameter *τ* in the cosine function describing the annual seasonality. Tick aggregation is equal to the parameter *k* in the negative binomial distribution. Estimates are maximum likelihood estimates for each sampling location. Study sites with significant trends are shown in bold.

**Table 3:** **Estimates for the parameters in the model describing the densities of questing nymphal plus adult *Ixodes ricinus *2006 – 2009**

**Sampling locations**	**Baseline tick density/activity**	**Population trend**	**Month of peak tick density/activity**	**Aggregation index**	**P-value**
**Appelscha**	**2.3**	**0.8**	**6.2**	**3.5**	**0.04**
Bilthoven	2.2	0.0	6.0	2.6	1.00
Ede	20.4	0.0	6.3	1.9	1.00
Eijsden	10.5	0.0	6.1	1.1	0.07
Gieten	8.5	0.0	5.9	1.9	0.61
**Hoog Baarlo**	**12.2**	**−1.6**	**5.7**	**2.2**	**0.02**
Kwade hoek	3.5	0.0	6.4	0.4	1.00
Montferland	11.0	0.0	5.7	0.6	0.08
Schiermonnikoog	2.5	0.0	6.1	1.8	1.00
Twiske	11.2	0.0	5.8	0.4	1.00
Vaals	5.6	0.0	6.5	0.3	1.00
Veldhoven	20.8	0.0	5.5	1.1	1.00
**Wassenaar**	**2.6**	**2.6**	**5.8**	**0.5**	**0.01**

See the legend for Table 1.

### Case study: longitudinal population sampling of ticks in duin and kruidberg

**Figure 4:**
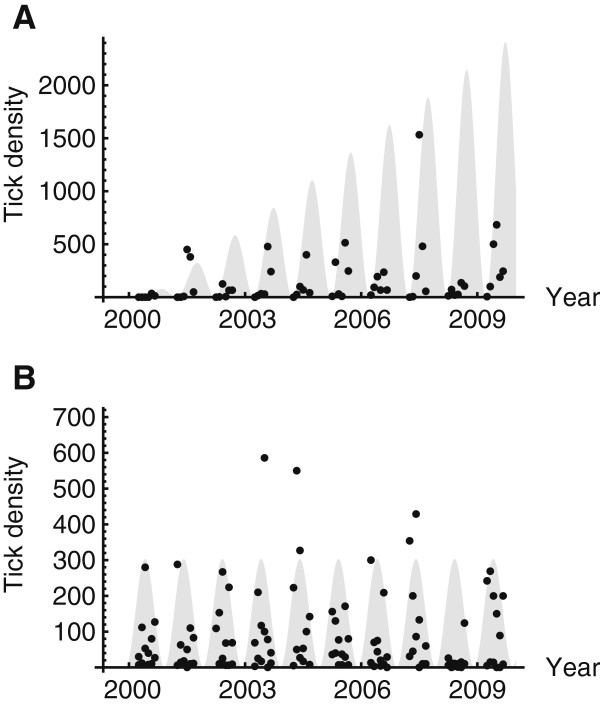
**Numbers of questing ticks at Duin and Kruidberg 2000 – 2009.** The population of larvae is shown in panel **A**. The population of nymphs plus adults is shown in panel **B**. A dot represents the number of ticks collected per drag (100 m^2^). Shaded area represents the best-fit for negative binomial distributions with the 95% confidence interval.

In Duin and Kruidberg, the density of ticks (sum of instars) increased during the last decade (*P*-value = 0.055). The abundance of larvae increased significantly at this location (P-value < 0.001, Figure 4A). In contrast, the density of nymphs and adult ticks did not increase (P-value = 0.9, Figure 4B). The abundance of small rodents was assessed in the vicinity of the tick-sampling sites (2000 – 2008). Four species were identified among the sampled animals: *Sorex araneus*, *Apodemus sylvaticus*, *Microtus arvalis*, and *Myodes glareolus*. The total numbers of these rodents per year were 1 (2000), 35 (2001), 34 (2002), 11 (2006), 6 (2007), and 45 (2008; see also: [49]). No significant change in the rodent population was observed in the sampling period. Abundance data of birds from this study area were not available.

Both roe deer and fallow deer (*Dama dama*) are present in Duin and Kruidberg. The population of roe deer has tripled from 200 in 1985 to 600 in 1997, and it has been stable since that time, varying between 400 – 600 roe deer per year. In contrast, in the same time period the population of fallow deer increased significantly. Only a few fallow deer were present in 1990 whereas the population of fallow deer in 2003 exceeded 800. Thus, since the start of our tick surveillance in Duin and Kruidberg in 2000, the total propagation capacity for ticks has steadily increased.

### Trends in the prevalence of ticks with *B. burgdorferi* sensu lato

A total of 372 out of 3104 nymphs and adults, collected between 2000 and 2009, tested positive for the presence of *B. burgdorferi* s.l. The prevalence showed seasonal variation (*P*-value < 0.001; see Additional file 5: Figure S3). A spatial variation in *Borrelia* prevalence was also detected (P-value < 0.001). The Borrelia prevalences of several study sites (Bilthoven, Ede, Eijsden, Gieten, Kwade Hoek, Montferland) were higher (annual mean 20%) compared to the other cluster of the remaining study sites (annual mean 8%). Only from one location (Duin and Kruidberg), long-term prevalence data were available: Despite seasonal and yearly variation, no significant increase or decline in *Borrelia* infection in questing ticks was observed (data not shown).

### Population dynamics of *B. burgdorferi* s.l

The most prevalent genospecies was *B. afzelii* (~65%), whereas *B. garinii*, *B. burgdorferi* sensu stricto, *B. spielmanii* and *B. valaisiana* were found less frequently (~10% each). *Borrelia lusitaniae* was identified for the first time in 2009 at one location.

**Figure 5:**
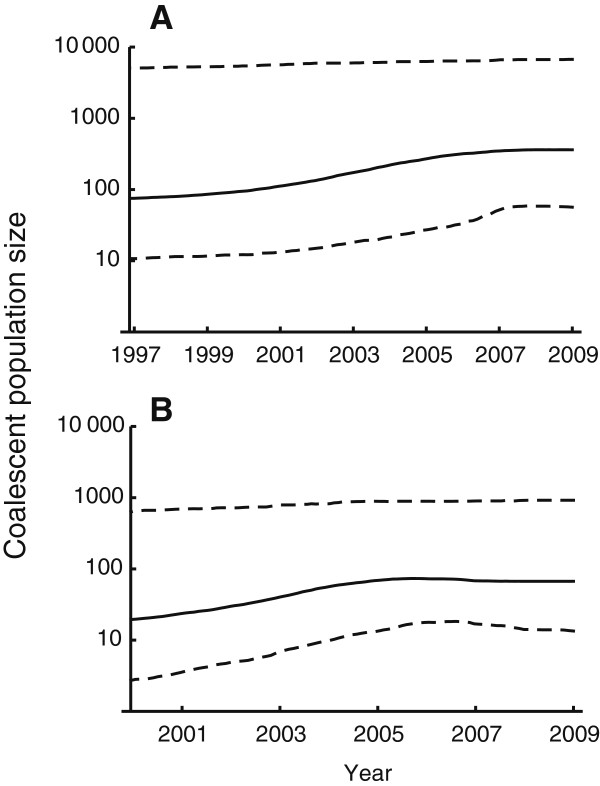
**Skyline plot of *B. afzelii *and *B. garinii *populations.** Changes in coalescent population sizes over years are shown in solid lines indicating *B. afzelii* (panel** A**) and *B. garinii* (panel **B**). Dashed lines enclose 95% credible interval.

A change in the genetic diversity of *Borrelia* over time is an indication that the sylvatic transmission of *Borrelia* in The Netherlands at large is changing. Negative estimates of Fs statistics indicate an excess of rare sequences due to a recent population expansion or genetic hitch hiking of *B. afzelii* (Fu’s Fs = −9.56, P-value = 0.02) and *B. garinii* (Fu’s Fs = −5.55, P-value = 0.03). The skyline plot appears to be more compatible with population growth within the last decade for *B. afzelii* and *B. garinii* (Figure 5) than an alternative explanation involving genetic hitchhiking.

## Discussion

Several independent analyses on various aspects of the tick cycle were performed in this study, which provide circumstantial evidence for a rise in abundance of questing infected ticks. In the period 2000 – 2008 the total surface area of habitats suitable for ticks increased by 19%. Although the actual tick density/activity was not measured in any of the newly arisen “high-risk” areas, it is expected that these areas are readily inhabited by ticks and their vertebrate hosts as they are generally adjacent to established tick-suitable areas.

The length of tick questing activity, based on the number of days above 7°C and 10°C per year since 1985, has increased. The onset of tick activity, as measured by the number of days above 7°C in the first half of the year, became significantly earlier over time. Here, only very simple and limited models were used. Other relevant climatic factors, such as saturation deficit, were not taken into account. Furthermore, climatic effects on the tick cycle as such, the vegetation, vertebrate hosts, diapause and human (leisure) activities were not taken into account either.

The long-term dynamics of vertebrate hosts is an indirect method for measuring changes in tick densities [21,26]. As the densities of tick hosts increase, it is to be expected that tick abundance will increase concomitantly. At least two principal sources of vertebrate blood for *I. ricinus*, namely the population of birds and deer have increased significantly in The Netherlands since the end of the last century. Also, a simultaneous increase of the predatory bird species barn owls as well as common buzzards was observed. Barn owl and common buzzard prey on small rodents and in this study are assumed to be indicator species for the nationwide rodent density [30,31]. Other factors, such as intensified nature conservation activities, may have resulted in the increase of birds of prey as well. The effect of host abundance on tick density is partially supported by our findings in the Duin and Kruidberg area. The density of larval ticks has increased significantly since 2000, which may be due to an increase in local populations of roe deer and fallow deer [41]. Remarkably, no simultaneous increase in nymphal and adult tick density/activity was observed in this area. Deer not only act as blood hosts for larvae and nymphs, they are the primary host for the adult deer tick and are key to the reproductive success of the tick [12,22,50]. Perhaps, an increasing abundance of deer resulted in a (much) better reproductive success of ticks, but only a minor/insignificant increase in the total number of feeding hosts.

Analysis of the total numbers of *I. ricinus* ticks collected in the 13 field sites between 2006 and 2009 showed an overall increase of 21% in the total tick-density/activity similar to what was observed in Duin and Kruidberg. The larval population attributed the most to the observed change in density, similar to that observed in Duin and Kruidberg. The 4% change in densities of nymphs and adult ticks may not be more than change variation. Densities of nymphs and adults may be more subject to density-dependence than larvae, which probably brought the densities back towards the long-term equilibrium. The massive reproductive potential of ticks allows larval numbers to vary widely, but become stabilized by density-dependent mortality at later stages [24].

As the majority of people acquire Lyme disease from infected questing nymphs and adult ticks, the observed limited increase in nymphal plus adult density/activity alone cannot satisfactorily explain the increase in tick bites and patients with erythema migrans. These direct measurements on tick density/activity seem to contradict the indirect measurements, such as the abundance of feeding and propagation hosts. The high spatiotemporal variation in tick densities/activities in combination with the relatively short-term measurement (~ 1 tick cycle) may be one explanation. Collecting data for longer periods may result in a more robust long-term trend analysis. Furthermore, the strongest increase in tick density/activity would have occurred in forest areas that arose between 2000 and 2008. Unfortunately, none of these areas were included in the longitudinal studies.

Another indirect method for measuring the human risk of acquiring Lyme disease is measuring the population dynamics of Borrelia in questing ticks. Using a population genetic approach, strong indications are found for recent population expansions of the two most abundant Borrelia species, *B. afzelii* and *B. garinii* in questing ticks, which are known to cause Lyme disease. These findings are explained best by an increase in tick abundance, as no indications were found that the fraction of ticks infected with *B. burgdorferi* s.l. altered in the Duin and Kruidberg region between 2000 and 2009. The three-year prevalence of *B. burgdorferi* s.l. has not increased at other locations in The Netherlands either [9], supporting our findings. Overall, increased tick abundance and increased Borrelia populations, without increase in Borrelia infection rate, can be explained by increase in the surface of tick- suitable areas.

The expansion of suitable habitats for *I. ricinus* concomitant with an uncontrolled increase in the roe deer population underscores the necessity to investigate how a sustainable natural environment can be maintained while minimizing the threats of tick-borne diseases in densely populated areas such as The Netherlands. These investigations should not only focus on Lyme disease, but also on other tick-borne pathogens [9,35,36,51-54]. Measures to reduce the size of the tick population, particularly in recreational areas, might be one sensible option to reduce the human risk for Lyme disease. Intervention measures may include habitat management, deer management and the introduction of biological control [55]. Alternatively, reduction of tick exposure may be facilitated by for example (temporal) disclosure of high-risk areas for the general public and by continued public information efforts focusing at prompt removal of attached ticks.

## Conclusions

Several biotic and abiotic factors that contribute to the overall abundance of questing infected ticks in The Netherlands have concomitantly increased in the past decades. Demographic analysis on genetic data from Borrelia in questing ticks supports these findings. Together, it supports the idea of an overall increase in abundance of questing infected ticks, and provides one explanation for the increase in reported tick bites and erythema migrans. The expected increase in the overall abundance of questing infected ticks was only marginally supported by longitudinal studies in 14 sites across the country between 2006 and 2009.

## Competing interests

The authors declare that they have no competing interests.

## Authors’ contributions

FG, AV, FJ, WT, JG organized and participated in the fieldwork for the collection and tick data. KT, AH, and HS collected and analyzed data on incidence of tick bites and Erythema migrans, land use, climate, and data on vertebrate abundance. MB, KT and HS collected and analyzed data on Borrelia infection rate and performed population genetic analyses. HS and KT drafted the manuscript and wrote the final version. All authors read and approved the final manuscript.

## Supplementary Material

Additional file 1 Table S1 Land usage types were assigned to three categories indicating habitats with probabilities for a high, low, or zero-tick density. Click here for file

Additional file 2 Figure S1 A: Number of days above 7 degrees Celsius since 1985. B: Number of days above 7 degrees Celsius since 1985 measured in the first half years (January – June). Click here for file

Additional file 3 Figure S2 Map of the Netherlands showing the 14 study sites where ticks were sampled. Click here for file

Additional file 4 Figure S4 Genetic divergence within and between *Borrelia burgdorferi* s.l. species based on the IGS-sequence. Click here for file

Additional file 5 Figure S3 Seasonal infection rates (fraction) of Borrelia *Borrelia burgdorferi* s.l. The study areas were separated into two groups: One group (black line) had an annual mean of 20% (Bilthoven, Ede, Eijsden, Gieten, Kwade Hoek, Montferland). The other group (dotted line) had an annual mean of 8% (Appelscha, Duin and Kruidberg, Hoog Baarlo, Schiermonnikoog, Twiske, Vaals, Veldhoven, Wassenaar). Click here for file
