# Supplementary material for: Circumstantial evidence for an increase in the total number and activity of borrelia-infected ixodes ricinus in the Netherlands
Source: Parasit Vectors. 2012 Dec 17;5:294. doi: 10.1186/1756-3305-5-294 (PMC3562265; doi:10.1186/1756-3305-5-294)
Supplement: Additional file 1 Table S1 — Land usage types were assigned to three categories indicating habitats with probabilities for a high, low, or zero-tick density. [file 1756-3305-5-294-S1.doc]

**Supplemental Material, Table1**

Land usage types were assigned to three categories indicating habitats with probabilities for a high, low, or zero-tick density.

| Land type | LGN code | Habitat type |
| --- | --- | --- |
| Corn | 2 | Zero |
| Potatoes | 3 | Zero |
| Beets | 4 | Zero |
| Cereals | 5 | Zero |
| Other crops | 6 | Zero |
| Greenhouse | 8 | Zero |
| Bulbs | 10 | Zero |
| Freshwater | 16 | Zero |
| Saltwater | 17 | Zero |
| Buildings in urban area | 18 | Zero |
| Bare soil in urban outskirts | 24 | Zero |
| Highways and railways | 25 | Zero |
| Open sand in coastal areas | 31 | Zero |
| Open drift and/or river sand | 35 | Zero |
| Tree nurseries | 61 | Zero |
| Fruit orchard | 62 | Zero |
| Agricultural grass | 1 | Low |
| Orchard | 9 | Low |
| Buildings in suburban area | 19 | Low |
| Forest in urban area | 20 | Low |
| Coniferous forest in urban area | 21 | Low |
| Grass in urban area | 23 | Low |
| Buildings in rural area | 26 | Low |
| Gras in suburban area | 28 | Low |
| Marshes | 30 | Low |
| Peat moor | 39 | Low |
| Other wetland vegetation | 41 | Low |
| Reed vegetation | 42 | Low |
| Forest in wetland | 43 | Low |
| Peat meadow | 44 | Low |
| Bare soil in nature area | 46 | Low |
| Heather | 36 | Low |
| Deciduous forest | 11 | High |
| Coniferous forest | 12 | High |
| Forest in suburban area | 22 | High |
| Dunes with low vegetation (<1m) | 32 | High |
| Dunes with high vegetation (>1m) | 33 | High |
| Heather dunes | 34 | High |
| Moderately grassed heath | 37 | High |
| Highly grassed heath | 38 | High |
| Forest in moorland area | 40 | High |
| Natural grassland | 45 | High |
