# Supplementary figures and images for: Circumstantial evidence for an increase in the total number and activity of borrelia-infected ixodes ricinus in the Netherlands
Source: Parasit Vectors. 2012 Dec 17;5:294. doi: 10.1186/1756-3305-5-294 (PMC3562265; doi:10.1186/1756-3305-5-294)

## Slide 1
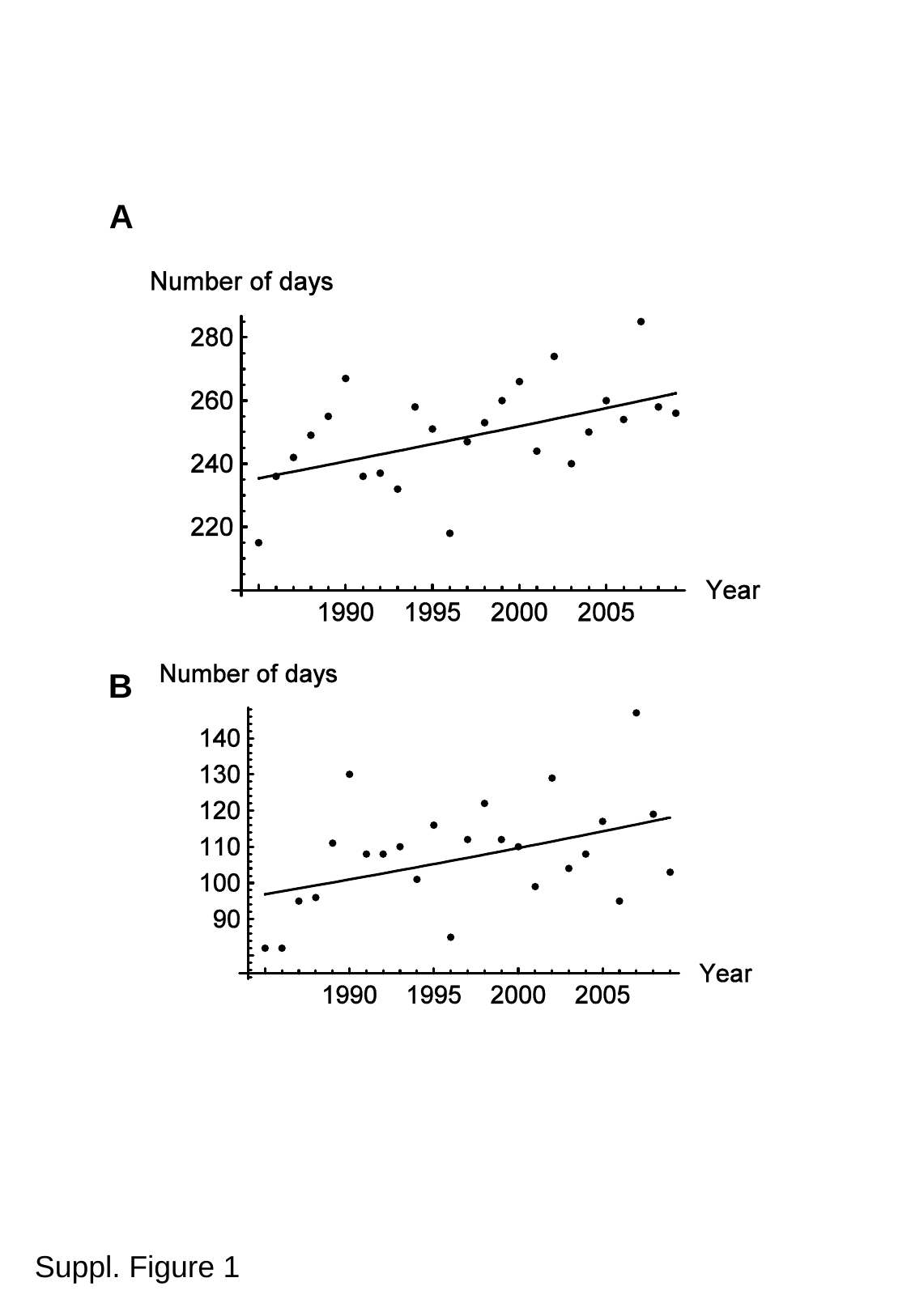

A
B
Suppl. Figure 1

Supplement: Additional file 2 Figure S1 — A: Number of days above 7 degrees Celsius since 1985. B: Number of days above 7 degrees Celsius since 1985 measured in the first half years (January – June). [file 1756-3305-5-294-S2.ppt]

## Slide 1
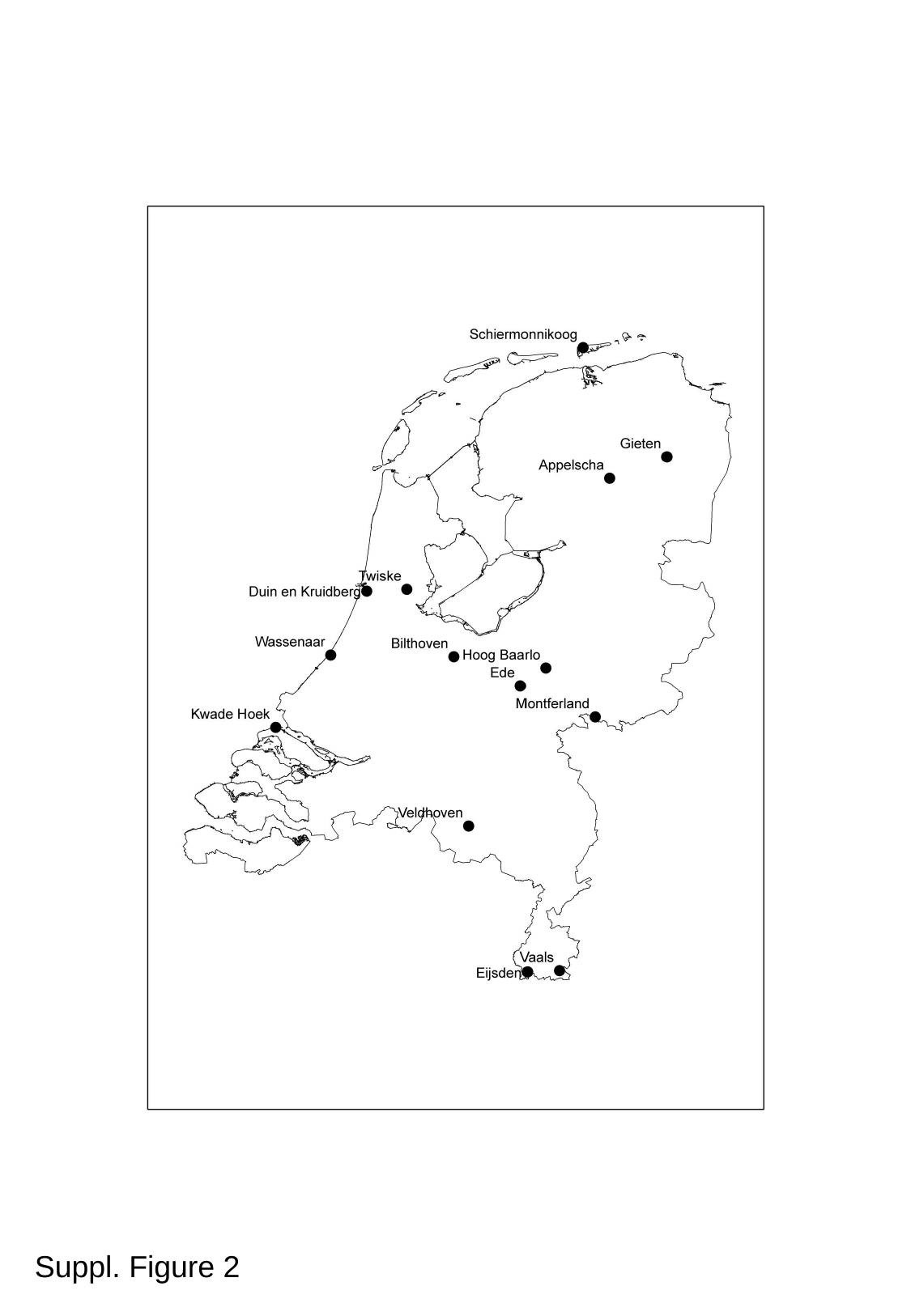

Suppl. Figure 2

Supplement: Additional file 3 Figure S2 — Map of the Netherlands showing the 14 study sites where ticks were sampled. [file 1756-3305-5-294-S3.ppt]

## Slide 1
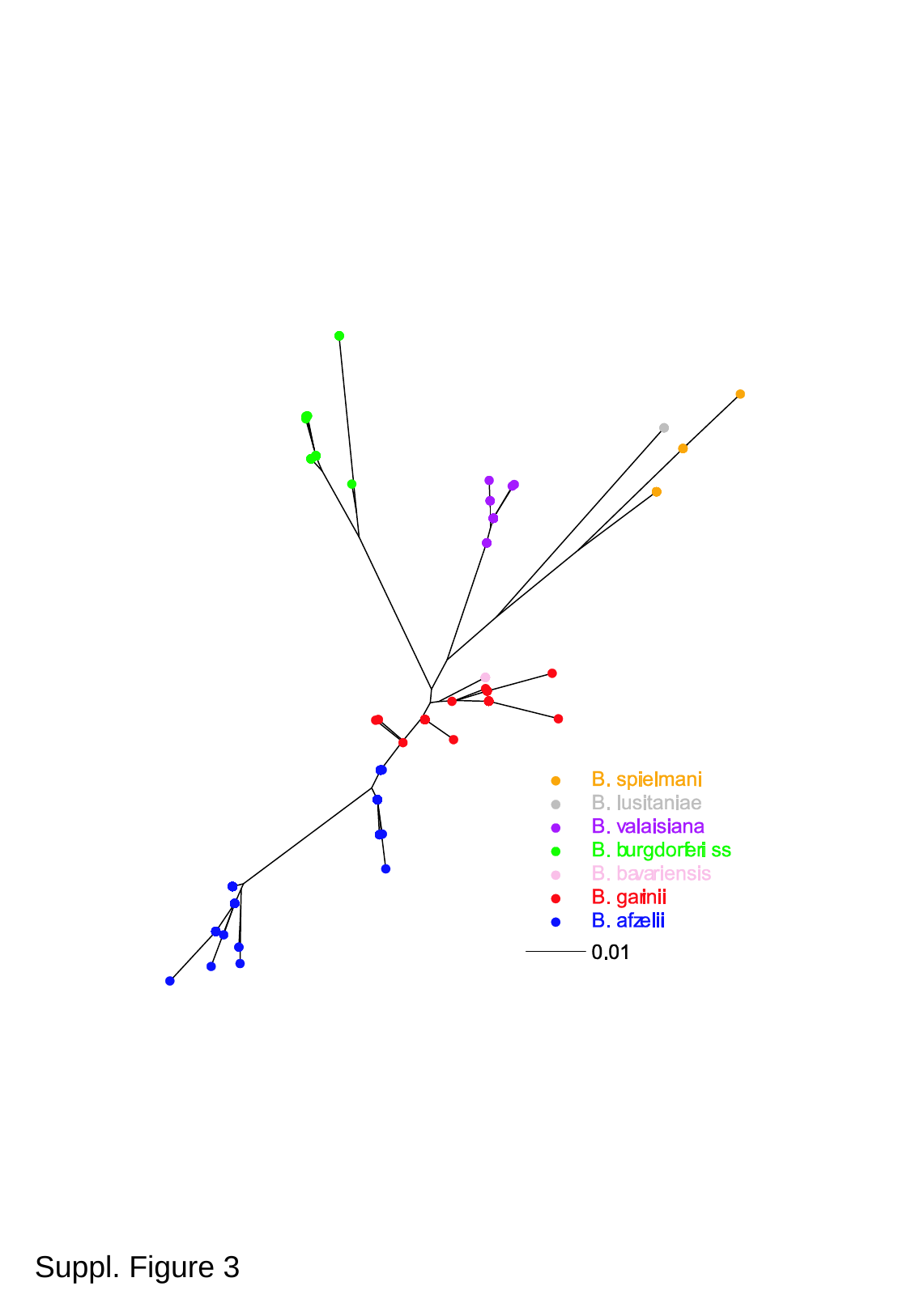

Suppl. Figure 3

Supplement: Additional file 4 Figure S4 — Genetic divergence within and between Borrelia burgdorferi s.l. species based on the IGS-sequence. [file 1756-3305-5-294-S4.ppt]

## Slide 1
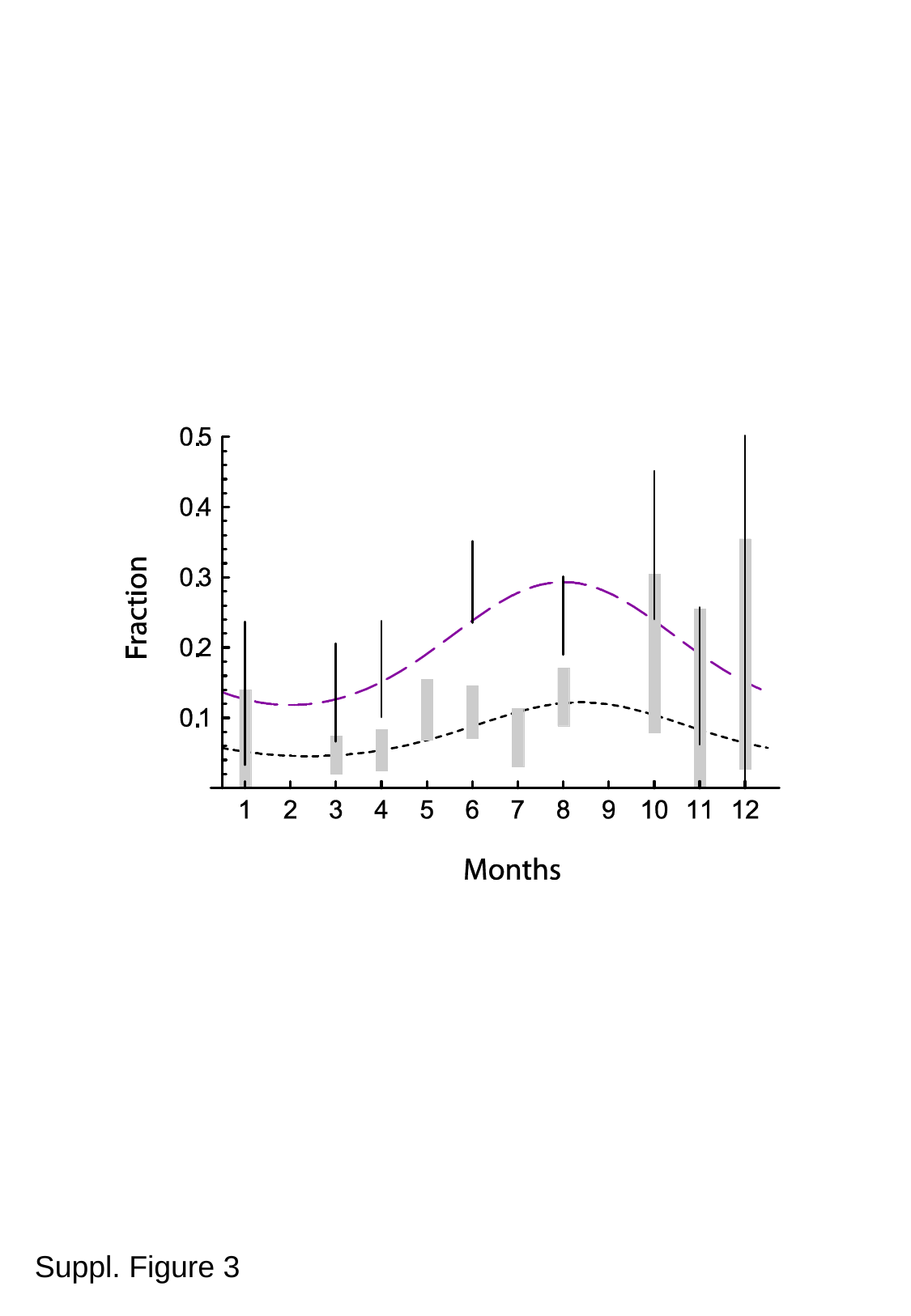

Suppl. Figure 3

Supplement: Additional file 5 Figure S3 — Seasonal infection rates (fraction) of Borrelia Borrelia burgdorferi s.l. The study areas were separated into two groups: One group (black line) had an annual mean of 20% (Bilthoven, Ede, Eijsden, Gieten, Kwade Hoek, Montferland). The other group (dotted line) had an annual mean of 8% (Appelscha, Duin and Kruidberg, Hoog Baarlo, Schiermonnikoog, Twiske, Vaals, Veldhoven, Wassenaar). [file 1756-3305-5-294-S5.ppt]
